# Hopital De Rovigo

**Published:** 1830-06

**Authors:** 


### HOPITAL DE ROVIGO.

### *Cure of a Vesico-Vaginal Fistula by the actual Cautery.**

On the third day after a natural but tedious labour, the patient in this case found that she had not the power of retaining her urine. Upon examination, several callous ulcers were detected near the vaginal orifice, which were successfully treated by anti-syphilitic means, and she was discharged in forty days. At the expiration of three months she returned to the hospital, still complaining of incontinence of urine, of which she confessed she had before only pretended to be cured, in order that she might return home. As it was suspected that there was a paralytic affection of the neck of the bladder, astringents, tonics, and other remedies, both general and local, were used, but without avail. Saturnine injections by the urethra suspended the involuntary discharge of the urine for a few days, but it returned.

Dr. Bellini again examined the patient per vaginam, and now ascertained that there was a small opening near the os uteri, through which the urine escaped. He at first believed that pressure upon the parts, by the head of the child, which had remained above the superior aperture of the pelvis for upwards of forty-eight hours during labour, might have occasioned this wound; but it was subsequently thought that the mischief had been produced by syphilitic ulceration. A mercurial treatment was adopted, and plugs of lint steeped in a solution of corrosive sublimate were passed into the vagina.

The involuntary flow of urine still continued, and Dr. B. was now convinced that it was necessary to destroy the callous edges of the wound, to give it a chance of healing. He introduced into the vagina a silver canula, which he guided with his finger; through the canula he passed a small iron rod, heated to a white heat, with which the edges of the wound were destroyed. The wound speedily healed, and the patient was entirely cured.

* Annali Univ.

